# Stroke Secondary to Air Embolism Following Laparoscopic Nissen Fundoplication

**DOI:** 10.7759/cureus.59168

**Published:** 2024-04-27

**Authors:** Angela Penney, Johann Park, Aimee Miller, Aryan Nasr, Ning Zhong, Forshing Lui

**Affiliations:** 1 Clinical Sciences, California Northstate University College of Medicine, Elk Grove, USA; 2 Neurology, Kaiser Permanente Sacramento Medical Center, Sacramento, USA

**Keywords:** nissen fundoplication, gas insufflation, laparoscopic surgery, stroke, air embolism

## Abstract

An air embolism is characterized by the entry of gas bubbles into the circulatory system, which can lead to the possible occlusion of blood vessels, posing a potentially life-threatening risk. While commonly associated with lung trauma or decompression sickness, it can also result from medical procedures such as central venous catheter insertion or, in our case, gas insufflation for laparoscopic surgery. We present the case of a 65-year-old female who suffered from a stroke secondary to an air embolism after undergoing a laparoscopic Nissen fundoplication in which carbon dioxide insufflation of the abdominal cavity was utilized. We also will discuss the elusive etiology of this complication as well as diagnosis, treatment, and proposed preventative measures.

A 65-year-old female with gastroesophageal reflux disease and a hiatal hernia elected to undergo a laparoscopic Nissen fundoplication for hernia repair. After a successful surgery, the patient was found with significant neurological deficits, including left-sided hemiplegia, numbness in the left hand, hemianopsia, dysarthria, and a National Institutes of Health Stroke Scale score of 20. CT head imaging revealed several low-density foci in the right frontal lobe, while CT neck and chest imaging revealed subcutaneous emphysema and pneumomediastinum. Subsequent labs were significant for an elevated lactate at 7.6 mmol/L. MRI of the brain depicted evidence of an acute infarct in the right frontal lobe with diffusion-weighted imaging (DWI) sequences. The imaging results were correlated with the patient's clinical presentation to establish the diagnosis of a nondominant hemisphere stroke, localized to an anterior branch of the right middle cerebral artery (MCA). After intubation and supportive treatment for three days, the patient was extubated and able to follow commands but had left facial weakness and diminished strength in the left upper and lower extremities. At the two-month follow-up visit, the patient no longer had any focal neurological deficits.

Air emboli, though very rare, can occur as a complication in laparoscopic surgeries that utilize CO_2_ for body cavity insufflation. Patients may be asymptomatic with small, self-limiting emboli, while others may exhibit pulmonary symptoms, cardiac arrest, or focal neurologic changes, depending on the emoji’s size and location. Given the wide range of patient presentations, the elevated mortality of laparoscopic procedures complicated by air emboli, and the rare occurrence of focal neurological symptoms as depicted in this case, rapid diagnosis and close postoperative observation and treatment are vital for both short-term and long-term patient outcomes.

## Introduction

An air or gas embolism occurs when air or gas bubbles enter the circulatory system and subsequently block the blood vessels distally or downstream. This potentially life-threatening condition is most commonly caused by lung trauma or decompression sickness in deep-sea divers. However, air emboli can also be iatrogenic or an inadvertent consequence of certain medical procedures, such as central venous catheter insertion, laparoscopic insufflation, lung biopsy, and cardiac or vascular surgery. The size and location of the emboli are key features that determine the severity of complications [1]. Small air emboli may quickly dissolve in plasma, spread into surrounding tissues, or enter pulmonary circulation and diffuse into the alveoli without leading to serious consequences. Such cases are usually asymptomatic or may have minor nonspecific symptoms such as lightheadedness, dyspnea, or chest pain [2]. However, larger air emboli can obstruct blood vessels and lead to ischemic changes in tissues and end-organ damage. Possible outcomes of large emboli specifically in pulmonary circulation may result in pulmonary hypertension, cardiac arrhythmias, hypoxia, and right heart failure. The patient may present clinically with jugular venous distention, pulmonary edema, and ST depression on electrocardiogram. Air emboli in patients with a patent foramen ovale (PFO) may cross from the venous system into the arterial system and potentially enter the brain. This can lead to headache, dizziness, seizure, stroke, vision loss, motor and sensory defects, or other neurological problems [3]. Air embolism is a specific subtype of gas embolism where air bubbles enter the bloodstream. Although these two terms are used interchangeably, it’s important to note that not all gas emboli are air emboli. This subtle distinction is important because different gasses have varying solubility levels in plasma. As a result, the persistence of the emboli may be different depending on the type of gas present in the patient’s circulation. This, in turn, can dictate the length of symptoms, severity of presentation, and patient outcomes. While air is composed primarily of nitrogen and oxygen gasses, which are both soluble in plasma, their solubilities are markedly less than that of carbon dioxide (CO_2_). For this reason, CO_2_ tends to dissolve more rapidly in plasma and is the preferred insufflation gas in laparoscopy [4]. Despite the general safety of this procedure, there have been reported cases of CO_2_ embolism with multi-organ dysfunction secondary to laparoscopic surgeries. In a 1997 meta-analysis of 489,335 laparoscopic procedures, only seven cases of CO_2_ emboli were documented (0.0014%). Unfortunately, two of these seven cases carried lethal complications [4]. We present a case of a 65-year-old woman who underwent laparoscopic Nissen fundoplication to repair a hiatal hernia. Soon after the procedure, she developed severe neurological symptoms which slowly subsided over days. This case may represent the second known case of cerebral arterial gas embolism secondary to laparoscopic Nissen fundoplication [5].

## Case presentation

**Figure 1:**
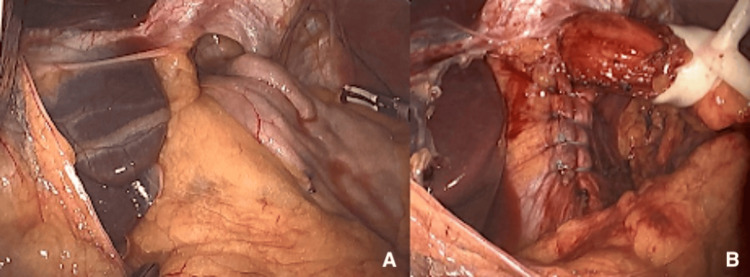
Images of laparoscopic Nissen fundoplication (A) before and (B) after the procedure.

A 65-year-old female with gastroesophageal reflux disease was evaluated and diagnosed with a 3-cm sliding-type hiatal hernia via endoscopy. The patient elected to proceed with laparoscopic Nissen fundoplication for hiatal hernia repair. The patient was taken to the operating room on the day of the surgery. The abdomen was insufflated with CO_2_ to 15 mmHg, and the Optiview trocar was placed with direct visualization, confirming no evidence of intra-abdominal injury. An 8 mm left subcostal port was placed and 5 mm ports were placed in the right upper quadrant and left lateral abdomen. The patient was placed in the reverse Trendelenburg position, and the abdomen was laparoscopically explored. The hiatal hernia defect was closed and a Nissen fundoplication was created by re-approximating the fundus to itself and the esophagus (Figures 1A-1B). Hemostasis was confirmed and there was no evidence of bleeding or bowel injury. The patient tolerated the procedure well and was transported to the post-anesthesia care unit (PACU) in stable condition.

**Figure 2:**
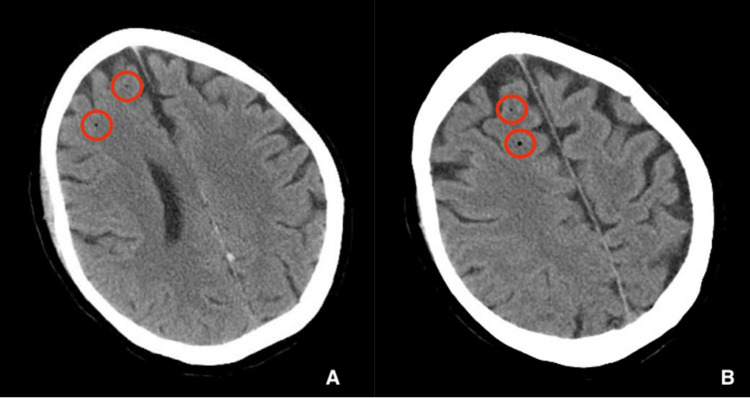
(A and B) CT head revealing two low-density foci (red circles) in two different CT sections of the right frontal lobe.

**Figure 3:**
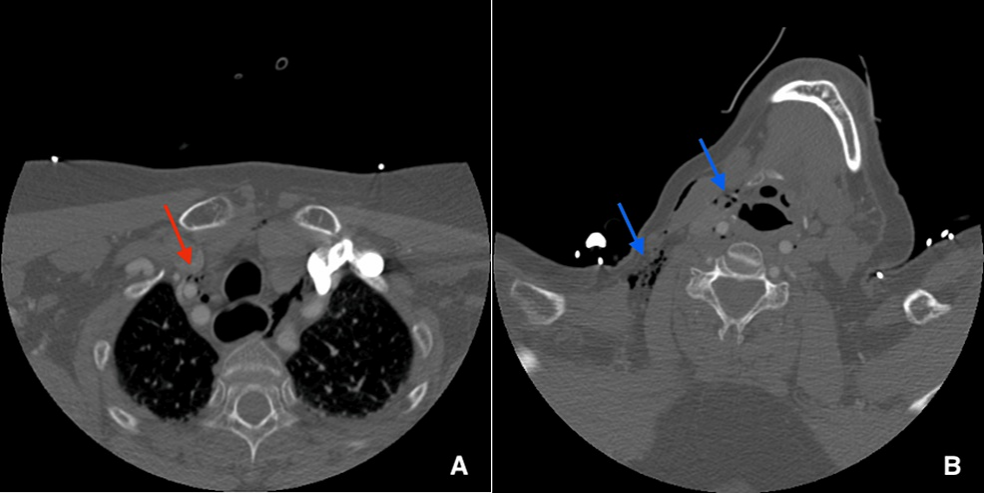
(A) CT chest displaying pneumomediastinum (red arrow); (B) CT neck with subcutaneous emphysema (blue arrows).

In the PACU, the patient was noted as *acting funny*, writhing, and not following commands. She was also unable to move the left side of her body. The patient was evaluated by Telestroke Neurology, who noted left-sided hemiplegia, numbness in the left hand, hemianopsia, dysarthria, and a National Institutes of Health Stroke Scale (NIHSS) score of 20. A computed tomography (CT) scan of the head was ordered, and while in the scanner, the patient experienced a generalized tonic-clonic seizure and vomited. She was intubated for airway protection. The CT revealed four low-density foci in the right frontal lobe (Figures 2A-2B) compatible with gas emboli. CT neck and chest revealed pneumomediastinum and subcutaneous emphysema (Figures 3A-3B). The patient was transferred to the intensive care unit (ICU). Labs included elevated lactate at 7.6 mmol/L, alanine aminotransferase (ALT) 47 U/L, aspartate aminotransferase (AST) 49 U/L, and alkaline phosphatase (ALKP) 128 U/L (Table 1).

**Table 1:** Significant serum laboratory values.

Test	Result	Reference range
Lactate	7.6 (high)	0.5-1.9 mmol/L
Alanine aminotransferase (ALT)	47 (high)	0-41 U/L
Aspartate aminotransferase (AST)	49 (high)	10-40 U/L
Alkaline phosphatase (ALKP)	128 (high)	37-117 U/L

**Figure 4:**
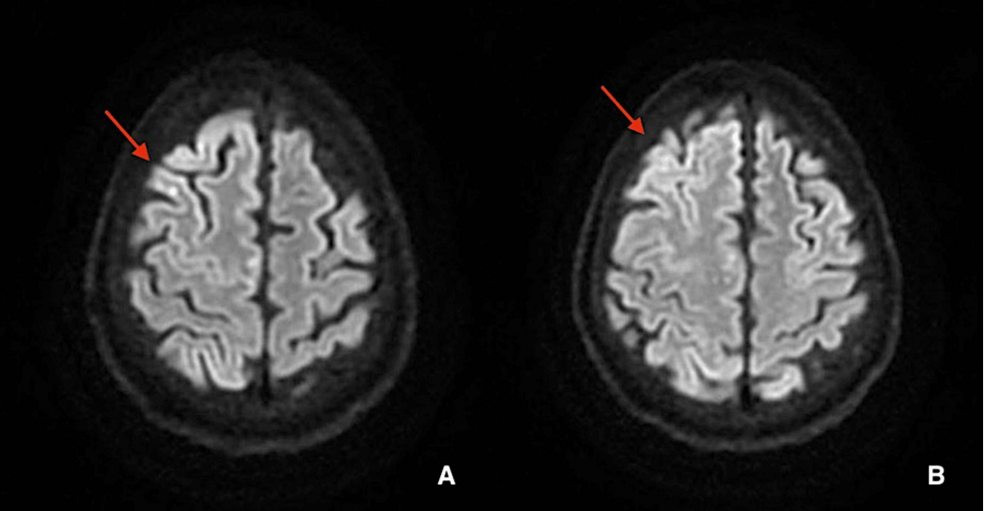
(A and B) Axial brain MRI images revealing right frontal lobe infarcts (red arrows). MRI, magnetic resonance imaging

On postoperative day 1, the patient remained unresponsive despite weaning off sedation. The electroencephalogram (EEG) showed a diffuse slow background recording with right hemispheric slowing and lateralized rhythmic discharges (LRD), suggesting an ictal pattern. The patient was treated with levetiracetam. Magnetic resonance imaging (MRI) of the brain showed evidence of an acute infarct in the right frontal lobe with diffusion-weighted imaging (DWI) sequences (Figures 4A-4B). The patient was successfully extubated on postoperative day 3. She was awake and following commands. She had left facial weakness and 3/5 strength of the left upper and lower extremities. Repeat head CT showed resolving gas emboli. On postoperative day 5, the patient demonstrated continued improvement with 5/5 strength of the left upper and lower extremities. She still stated she was not able to think very well due to *brain fog*. 

The patient was examined postoperatively at the two-month follow-up neurological visit via video, and no focal neurological deficit was detected by the neurologist.

## Discussion

Air embolization is a rare complication of laparoscopic surgical procedures, particularly when utilizing CO_2_ gas for abdominal cavity insufflation, and occurs when air enters the circulatory system, leading to obstruction of major blood vessels. This obstruction induces ischemia and subsequent organ damage. It may also be associated with seizures, headaches, dizziness, and visual field deficits [5] along with possible injury and inflammation to the vascular endothelium. While gas emboli are already a rare complication, those traveling to the cerebral vasculature, as observed in our patient, are even rarer and can precipitate a stroke or transient ischemic attack (TIA). The reported incidence rate of gas embolism following laparoscopic procedures is as low as 0.15%, with an estimated mortality rate as high as 30% [6]. Kulkarni et al. reported a similar case of cerebral arterial gas embolism secondary to laparoscopic Nissen fundoplication [5] and, to our knowledge, ours could represent another similar case. 

The etiology of this phenomenon remains elusive. In the setting of hemodynamic compromise, such as intraoperative vessel trauma, air may be directly introduced into the bloodstream, embolizing through the venous system. This typically necessitates a pressure gradient created by initial CO_2_ insufflation, overcoming the lower pressures of the venous system, and pushing gas into the blood vessels. There have also been reports of gas embolism causing acute ischemic strokes after accidental needle sufflation into the liver. Interestingly, our patient’s operative report indicated no evidence of intraoperative injury or excessive bleeding and no evidence of liver injury following retractor removal, raising suspicions about how air infiltrated the bloodstream.

Another critical question surrounds discerning how the emboli reached the cerebral vasculature. Following the anatomic sequence of blood flow, the emboli must have traversed from the venous system surrounding the abdominal cavity to the cerebral arterial system without passing through the smaller capillaries of the pulmonary system. Possible explanations include the presence of an anatomical anomaly, such as a PFO or cardiac septal defect, allowing air to bypass the pulmonary vasculature and embolize the cerebral arterial system. Another possibility is abnormal arteriovenous connections within the pulmonary vasculature, known as sperr-arteries [7,8]. These arteries, reported in the literature to cause paradoxical emboli in patients without intracardiac defects, remain a plausible consideration in our patient, given the absence of documented evaluation for a PFO or other cardiac septal defect.

Our patient was diagnosed with a nondominant hemisphere stroke, localized to an anterior branch of the right middle cerebral artery, during postoperative observation after the discovery of focal neurologic deficits, such as left-sided hemiplegia, left-hand numbness, hemianopsia, and dysarthria. Subsequent CT (Figure 2) and MRI (Figure 3) imaging confirmed these findings with four low-density foci and evidence of an embolic infarct in the right frontal lobe with mild loss of gray-white differentiation, respectively. Reliable detection of intracardiac gas, which would be expected sequelae if air has embolized from the venous system, can also be done through transesophageal echocardiography and transesophageal, transcranial, or precordial Doppler ultrasonography. However, these measures are rarely performed preemptively unless there is a predetermined elevated risk in the patient [8]. 

Regarding treatment, the patient made a rapid recovery of neurological function within a few days postoperation without specific medical interventions targeting the embolism, suggesting the potential for self-resolution as the gas absorbs into the tissues. Nevertheless, preventative measures may mitigate the likelihood of a gas embolism during laparoscopic surgeries. One such recommendation is to ensure the trocar is positioned fully inside the abdominal cavity before insufflation, confirmed by Veres needle aspiration, or test inflating a small volume of CO_2_ before full initiation of insufflation. Lower CO_2 _insufflation pressures, specifically below 10-15 mmHg, and maintaining higher central venous pressure in the patient through adequate intravenous (IV) fluid replacement pre- and intraoperatively, have also been associated with reduced chances of gas embolization [9,10]. Finally, positioning the patient in Trendelenburg may theoretically counteract the buoyancy of gas bubbles in the bloodstream, preventing their travel to the brain. However, this measure remains insufficiently studied and the needs of the surgical procedure being performed will likely determine patient positioning.

## Conclusions

Air emboli have many causes such as lung trauma, barotrauma, central venous catheter insertion, and, as in the case of our patient, laparoscopic surgery. Air emboli following laparoscopic procedures are rare postoperative complications following CO_2_ insufflation of a body cavity, which can lead to a wide range of signs and symptoms dependent on emboli size and location. Due to the wide range of patient presentations, the relatively high mortality of laparoscopic procedures complicated by air emboli, and the rare clinical presentation of focal neurological symptoms, as described in this case, rapid diagnosis and appropriate postoperative observation and treatment are crucial for the patient's short-term and long-term outcomes.
